# An ethnographic study of improving data collection and completeness in large-scale data exercises

**DOI:** 10.12688/wellcomeopenres.14993.1

**Published:** 2019-12-16

**Authors:** Mary Dixon-Woods, Anne Campbell, Emma-Louise Aveling, Graham Martin

**Affiliations:** 1 THIS Institute, Department of Public Health and Primary Care, University of Cambridge, Cambridge, CB2 0AH, UK; 2 Faculty of Medicine, Imperial College London, London, SW7 2AW, UK; 3 Harvard T. H. Chan School of Public Health, Boston, Massachusetts, 02115, USA

**Keywords:** Registries, Ethnography, Health professions, Data collection, Lung cancer, Abdominal aortic aneurysm, Surgery

## Abstract

**Background: **Large-scale data collection is an increasingly prominent and influential feature of efforts to improve healthcare delivery, yet securing the involvement of clinical centres and ensuring data comprehensiveness often proves problematic. We explore how improvements in both data submission and completion rates were achieved during a crucial period of the evolution of two large-scale data exercises.

**Methods:**  As part of an evaluation of a quality improvement programme, we conducted an ethnographic study involving 90 interviews and 47 days of non-participant observation of two UK national clinical audits in a period before submission of data on adherence to clinical standards became mandatory.

**Results: **Critical to the improvements in submission and completion rates in the two exercises were the efforts of clinical leaders to refigure “data work” as a professionalization strategy. Using a series of strategic manoeuvres, leaders constructed a cultural account that tied the fortunes of the healthcare professions to the submission of high-quality data, proposing that it would demonstrate responsibility, transparency, and alignment with the public interest. In so doing, clinical leadership deployed tactics that might have been seen as unwarranted managerial aggression had they been imposed by parties external to the profession. Many residual challenges were linked not to principled objection by clinicians, but to mundane problems and frustrations in obtaining, recording, and submitting data. The cultural framing of data work as a professional duty was important to resolving its status as an abject form of labour.

**Conclusions: **Improving data quality in large-scale exercises is possible, but requires cooperation with clinical centres. Enabling professional leadership of data work may offer some significant advantages, but attention is also needed to mundane and highly consequential obstacles to participation in data collection.

## Introduction

Data systematically collected across multiple settings have an important role in revealing unwarranted variations in healthcare practices, processes and outcomes and in identifying areas for improvement ^1– 4^. Purposefully designed large-scale exercises involving data extraction from medical records into standardized formats are now well-established in many health systems. Such exercises vary in the specifics of their design (for example in using census or sample methods) and go by different names, including registries and clinical audits. Their unifying feature is that they require participating clinical centres to prepare information on specific measures (usually by reviewing clinical records or prospectively establishing data collection systems) using standardized definitions, and then submit the data to a central register using a standardized template to enable comparison of performance ^5^. These kinds of large-scale data exercises are often associated with improvements over time ^6– 9^, but they are not straightforward to design or execute.

One challenge is that they rely for their effectiveness on the accuracy, reliability and completeness of the data ^10^, yet missing data and variable participation rates between organizations are frequent problems worldwide ^11, 12^. Discrepancies, sometimes substantial, between what is recorded in routine medical records and what is reported to registries/audits are frequently found ^13, 14^. Efforts to improve data quality have been varied: they have included technological innovation based on electronic health records, data linkage and “big data” strategies, financial incentives, and exhortations for improved accuracy. None has been fully effective ^15, 16^, and methods of quality assurance remain resource-intensive ^17^. Means of addressing these challenges are much needed, but will depend on better understanding of what influences data completion and quality in clinical centres and how they may be addressed. In this article, we respond to this need, reporting a qualitative study of large-scale data collection using case studies of two UK national clinical audits during a crucial period in their evolution when they demonstrated improvements – though not perfection – in data completeness and quality.

Some context is useful. In the UK, large-scale clinical audit originated as an activity largely led by the medical profession. An early example was the monitoring of survival rates after cardiac surgery through voluntary submission of data to the Society of Cardiothoracic Surgery, which began in 1977, initially through the collection of data aggregated at the level of surgical units, and later through submission of patient-level outcome data. In the early years of many audits, participation by National Health Service (NHS) organizations, while widespread, was voluntary, and often depended on local professional enthusiasm ^18^, but audit has evolved into a large-scale activity organized on a national scale. Just under half (~30) of the current ~70 national clinical audits are operated by professional groupings (such as the royal colleges or professional societies) as part of a national managed scheme supported by government funding: the National Clinical Audit and Patient Outcomes Programme (NCAPOP) ^3^. Participation in NCAPOP audits is now mandatory for NHS organizations, following the introduction of a contractual requirement in 2012.

Data from the audits are pluripotent and mobilized for many goals, including quality assurance, pay-for-performance schemes, and inspection and regulation. As a result, large-scale audits have assumed a somewhat hybrid character: though mostly still led and organized by professional groups, they are funded by government and are part of the system’s performance management architecture.

The trend for appropriating audit data as a mechanism for external rather than internal control of professional work is evident globally. In the United States, for example, quality measures and data collection activities that originally developed within professional structures are increasingly repurposed as mechanisms of transparency and accountability ^19^. However, the consequences of these shifts in professional ownership for data collection practices remain poorly understood. One possibility is that the apparent co-option of large-scale data exercises such as clinical audit might, rather than improving data quality, provoke professional resentment and disengagement, degrade the perceived value of audit as a professional learning resource, and activate familiar dysfunctional effects of performance measurement, such as gaming of data ^20^. Few studies have, however, explored this or the other influences on data collection and submission ^21^.

In this article, we examine influences on data submission and completion by clinical centres using two large-scale UK clinical audits: the National Lung Cancer Audit (“the Lung Audit”) and the National Vascular Registry (“the Vascular Registry”). Both share histories characteristic of clinical audit in the UK more generally. Though initially founded to serve as voluntary intra-professional endeavours, both have increasingly assumed features of performance management.

## The two audits

What we term the Lung Audit had its origins in 1999 in the efforts of an informal group supported by the Royal College of Physicians (RCP), itself a peer-led statutory body. The Lung Audit was formally established in 2004. By 2009, it had been implicated in the substantial improvements in clinical care that occurred during this period ^22, 23^, achieved by identifying variations and inequalities in care processes and outcomes and targets for improvement. Data submission was initially voluntary, but all NHS organizations in the UK responsible for diagnosing and treating patients with lung cancer were, from 2012 onwards, required to upload their cases under a new contractual requirement to contribute to NCAPOP audits.

The Vascular Registry (which has had various names) was initially formed in 1997 by a “few interested surgeons pooling data from different centres to provide a comparison of outcomes in vascular surgery” ^24^. Like the Lung Audit, it has evolved over time, initially voluntary and becoming more formalized and organized, while still led by the Vascular Society of Great Britain and Ireland (a professional society for advancement of quality, training and research in vascular health, led by vascular surgeons themselves). Data submission to the audit has been mandatory since 2014 when the Registry entered the NCAPOP program.

In common with many large-scale data exercises, the Lung Audit and the Vascular Registry experienced problems with obtaining reliable and complete contributions of data from their inception. Some centres submitted high-quality, complete data on time; others none at all; some were inconsistent in submission or contributed data that were neither complete nor clean ^22, 25^. Improvements in both audits have occurred over time, with the most recent reports suggesting generally high completion rates, though also variability in completion of some fields and ongoing variation in returns between centres ^26, 27^. The Lung Audit, in its most recent annual report, notes recording of cancer stage for 95% of patients, for example, but much weaker completeness for others fields (e.g. performance status, recorded for only 76% of patients, with individual hospitals ranging from 0% to 100% data completeness) ^27^. Similarly, the Vascular Registry’s most recent annual report reports case-ascertainment rates of around 90% for most procedures, but much lower rates for some (e.g. endovascular lower limb procedures, 27%), and significant variations in submission rates between centres ^26^.

One way of explaining the overall improvement over time is simply to note that data submission, which had been voluntary when the audits were first launched, became mandatory (2014 for the Vascular Registry; 2012 for the Lung Audit). The Lung Audit also changed its methods of data collection in 2014, and in 2015 began supplementing data submitted by trusts with other data sources. Yet substantial improvements were occurring both in submission rates and accuracy long before the introduction of the contractual requirement ^28, 29^, suggesting that mechanisms other than compulsion were at work. Further, the persistent variability in completion of some data fields suggests that neither mandating data collection nor technical innovation provide a full solution.

An opportunity to examine what else might explain the observed improvements in data collection in the audits prior to the mandating of data collection—and what might inhibit further gains—was presented by the involvement of both audits in a quality improvement programme. Known as Closing the Gap through Clinical Communities, it was organized and funded by the Health Foundation, a major charitable foundation. The Lung Audit and the Vascular Registry each participated in quality improvement projects in the programme, and alongside goals relating to improving quality of care in their clinical areas, both set improving quality of data submission as a key objective of their improvement activities. As demonstrated by their annual reports, both audits saw continued improvements over the course of the programme in both data submission and data quality ^26, 27^. We use our study to explore how these improvements were achieved in a crucial period of the audits’ evolution, before data collection became mandatory.

## Methods

As part of an evaluation of the Closing the Gap through Clinical Communities programme (November 2009-May 2012), we conducted an ethnographic study involving non-participant observation, interviews and documentary analysis of the quality improvement projects linked to the Lung Audit and the Vascular Registry. We conducted 90 semi-structured interviews (45 in each project with key programme personnel and those involved with the programme at participating sites), as well as 25.5 days of observations in the lung cancer project and 21.5 days in the vascular surgery project. Participants were identified through lead contacts either at the programme or the programme sites, and invited to take part using a package of materials that explained the study.

Interview prompt guides covered individuals’ roles in the quality improvement project, understanding of the aims of the project, strategies for achieving the aims, challenges anticipated and experienced, aspects of the project that were most and least successful and the factors that contributed, changes made to the project to respond to difficulties, unexpected outcomes, and what might have been done differently.

Interviews were audio-recorded, with written informed consent provided in advance of interviews, and transcribed verbatim. Fieldnotes from observations were de-briefed within the team, audio-recorded and transcribed. Relevant project documents, including project plans, reports and training materials, were also collected and analysed.

Data analysis was based on the constant comparative method ^30^. The transcripts and fieldnotes were first read and reread, resulting in an initial broad-brush coding of all sections of interviews and observations relating to the collection, measurement and use of data. Then, facilitated by NVivo 9 software, more detailed codes were iteratively developed and applied. This process highlighted the significance of the themes of the shifting ownership and purpose of the audits, and the challenges of valid data collection and submission. These themes informed a final analytic stage involving a more theoretical coding process in which these categories were further refined, informed by relevant theoretical and empirical literature, and discussed and agreed by all co-authors.

The study was given a favourable opinion by the Leicestershire, Northamptonshire & Rutland Research Ethics Committee 1(10/H0406/77).

## Results

Our analysis identified the importance of two influences on participation and completion rates and quality of data submitted to the two audits. The first was strategic action by professional leaders who sought to promote collective responsibility for the quality of care provided by their peers. The second was mundane interferences in collecting and submitting data.

### Contributing data as the exercise of professional mandate

Professional leaders in two audits were faced with the challenge of persuading their (often reluctant – and sometimes truculent) peers to cooperate with collecting and submitting high-quality data in a policy context that seemed increasingly inclined to weaponize data as a means of blaming and shaming ^31^. Our analysis suggests that one major reason for the improvements seen in quantity and quality of data submission in this context was the highly purposeful efforts of professional leaders to refigure what we term “data work” as a professionalization strategy ^32^. Leaders achieved this through the use of cultural framing to convince providers that full and authentic participation in the audit made sense and was “consistent with their own basic interests or identities” ^33^, (p9) while also linking those interests and identities to a collective purpose. They therefore produced what Kieran Healy terms a “cultural account” ^34^ – a set of institutionalized stories, ideas and values about the nature and meaning of an activity. The success of this approach depended on persuading peers that audit data was both a necessary part of clinical practice and a professional responsibility, and that benchmarking and cross-centre comparison data should – even as it restricted individual autonomy – be regarded as a positive development for the profession as a collective entity. Our observations at events organized by the two projects found, for example, that leaders sought to demonstrate that collecting and submitting data was fully aligned both with professional values and with the wider public interest. By claiming custody of those values, leaders sought to show that contributing data was not only in the interests of improvement but also consistent with a positive professional identity and values.

*Ensuring good quality data input into that is part of achieving consistency, so if you like, it’s about professionalizing people’s attitudes, so the data itself is important but actually getting people to understand that putting in good quality data is important, and embedding that in part of their daily activity. [Vascular Registry: project lead]*

One important tactic used by professional leaders was the deliberate and skilful presentation of the external pressures for accountability as an opportunity to strengthen internal professional mission. Leaders promoted the view that demonstrating their profession’s ability to conduct high-quality clinical audit helped (rather than hindered) efforts to retain self-governance. For Eliot Freidson ^35^, this kind of control over the content of work and the standards that should apply to that work is a defining feature of professionalism. This was particularly true for the Vascular Registry, where the professional society was quite explicit in published documents and in interviews that its running of the audit allowed it to demonstrate both its professional values and commitments. Its leaders stressed the risk that if the profession failed to get its house in order, the mandate for data collection would be seized by others, with deleterious consequences both for the profession and its individual members. More positively, leaders sought to show how invoking the trustworthiness of professional groupings could help to advance professional agendas, allowing them to conduct their affairs in ways that were consistent with modern forms of governance, while also maintaining professional control.

*Critics argued that the poor results for elective AAA [abdominal aortic aneurysm] surgery were a reason to delay the introduction of AAA screening, since there is no point in identifying men with an AAA unless the services to treat them are optimal. The Department of Health was, however, reassured by the response of the VSGBI [Vascular Society] and gave screening the go ahead* ^36^. *[Extract from document, Vascular Registry]*

As part of the strategic mobilization of their peers, professional leaders also sought to convince their colleagues that an alternative to clinical audit – using routine administrative data from medical records – would most likely misrepresent their activity and outcomes, owing to known problems with these data.

*The problem we have with HES [Hospital Episode Statistics compiled using routine administrative data] is the information was never accurate enough for making clinical decisions. […] I did an audit on the HES system against our own records of the lung cancer patients. […] There was about 15% at one end of people who were diagnosed with lung cancer [on HES] that didn’t have it, and at the other end there was 15% of people who were being missed who had got lung cancer but had been recorded on HES as having something else.” [Lung Audit: coordinator]*

Leaders in both audits emphasised the reputational risks for individual sites associated with non-submission of data and of failure to ‘volunteer’ them for national publication. They pointed out that the risks accrued not just to individual sites but also affected the integrity and standing of the audits as a whole. Thus, the professional leadership sought to mobilize cooperation by binding the fortunes of the audit at a collective level to those of individual actors.

*There is an urgent need for clinicians to improve contribution to national audit. Without the ability to accurately measure what we do, we are unable to describe how we need to change, or what change we are achieving. Surgeons used to be able to say that their practice was good. Recent publications have given the lie to this. National clinical audit will allow clinicians to reclaim the right to advise patients from a clear understanding of the quality of service that they provide. Audit needs to be a central part of our culture. [Document extract, Vascular Registry] (p50)* ^37^

Similarly, with the Lung Audit, participants invoked arguments that combined appeals to improving quality of care with defence of professional autonomy.

*[Collecting data on cancer staging is] particularly important for this hospital and this bit of the world because we always have low surgery rates and low rates of radical treatment, and the argument is that the reason we have that is because our patients present much later and they’re much sicker. But you have to have evidence to support that otherwise people think you’re just not doing a very good job. [Lung Audit: respiratory physician]*

Despite some initial resistance and criticism of the push for better data on the part of the clinical centres, improvements did occur. In the Vascular Registry, for example, the rate of submissions nationally increased from 380/month to 475/month over the period of the quality improvement project (similar data in this form are not available for the Lung Audit). In both the vascular and the lung cancer communities, it became broadly acknowledged that failure to produce credible data could result in unwarranted perceptions of weak performance both collectively and individually.

*Well, from a purely selfish point of view, as we have good results, I like to let the world know we have good results. On the other hand, it’s also good to feed our results into the National Vascular Database so that they can show the world that we have good results. [Vascular Registry: hospital-based vascular surgeon]*

For vascular surgeons, the perceived value was profoundly influenced by the wider context. They accepted that it was inevitable that their data would be made public (by centre if not by surgeon), in part because it had already happened in other, closely related areas of surgery (cardiac surgery, for example), and in part because the policy context meant doing the data work was essential to gaining recognition as an accredited centre for AAA screening. Producing the data – and demonstrating the quality of the service provided – was thus presented by leaders, and eventually became widely understood locally, as an indispensable task. Local and national contexts were highly interdependent in this sense, with implications for professionalism and the professionalization of data entry. Thus, while the coercive pressure associated with performance management was not unimportant, it was the combination of this with the sense of professional direction that seemed to be especially powerful in motivating action at the level of the contributing centres.

*The two things that are driving us is that to be a screening unit we have to do it, and then this central push from the Vascular Society. [Vascular Registry: vascular surgeon]*

Critical to establishing this cultural account of clinical audit was the legitimacy that the professional leadership “breathed” ^38^ into the enterprise. This feature also enabled the leadership to deploy tactics to encourage better data submission that might otherwise have been resisted as illegitimate managerialist aggression. For example, both audits began to use “traffic light” systems to classify contributing organizations. In vascular surgery, centres were rated against criteria including data completeness and coding errors, with the information reported quarterly to senior administrators at each organization ^39^. The Lung Audit similarly classified centres on their data contribution and the achievement of specific targets. But those leading the audits also restrained themselves from applying too much of a hard edge:

*We’re pushing but actually, you can push too hard, you can be too assertive, you can be too didactic and what you’ll do, you’ll do a number of things: you’ll put people’s backs up, so you’ll get disengagement; you will miss things that are important because you’re too busy focused on the road and not able to look up and just look around and pause. [Vascular Registry: clinical lead]*

To the extent that they maintained professional autonomy at the collective level, and operated through modes such as peer influence and top-down pressure *within* professions, such tactics were by-and-large accepted by the participating sites. Persuasive tactics on the part of professional leaders were important in producing this broad cultural frame. But also vital to ensuring that the audits were not rejected in clinical centres as blunt tools of national accountability was further creative and skilful work by local actors to present data work as an instrument for improving local practice, including better understanding of the relationship between care processes and patient outcomes, and challenging colleagues who had previously been intransigent to efforts to change their behaviour.

*I spent hours going through it all, and our surgical rate’s dropped to 7% and that’s well below the national average [in the Lung Audit] and I wanted to know why. So that’s the other thing I’ve been doing, is going through all the patients to work out why, and it turns out that actually having the performance status and the staging has made a massive difference. [Lung Audit: hospital-based respiratory physician]*
*I went and looked at every aneurysm that had been operated on, to see if they’d had the traffic light forms filled in, to see if they’d been discussed at an MDT, to see if they’d been seen by an anaesthetist and that kind of thing. And the results were fairly poor. [...] Whereas now we’ve got an anaesthetic pre-assessment, every patient is discussed at the MDT. So I think when we first did the audit, because the results were so poor, that’s when we were like, “What is our priority here and how can we do it”? [Vascular Registry: hospital-based vascular surgeon]*

### The mundane troubles of data collection

Though improvements in rates of contributions of data to the audits occurred throughout our study, variability between clinical centres remained: the cultural account of clinical audit as a valuable activity linked to professional interests and local gains was not always enough to achieve the improvements in data quality that audit leaderships sought. To secure the supply of data also required addressing the troubles of data collection.

Some of the trouble lay in ongoing fault-finding among professional peers about the work of data, including, in particular, the data fields they were asked to complete: some were seen to lack clinical value, while the omission of others was said to result in the neglect of important aspects of practice. This was a problem anticipated by Garfinkel’s argument that the categories provided to clinicians as rules to report their clinical practice may “distort the reality” of that practice so that clinicians may “resent it or otherwise suffer it” ^40^ (p196).

*[She] says, “The boxes aren’t the kind of boxes that you want.” And the oncologist, he says the same thing: “This form wasn’t designed by a clinician because they aren’t the boxes you would like.” [Lung Audit: observation]*
*Whether you see the patient’s relatives or not, it’s never been counted and published […] I think it is wrong. [Lung Audit: respiratory physician]*

A second trouble was more mundane in character, but more consequential in terms of its impact on rate of data collection and quality of data: the tedious, time-consuming and exasperating nature of the tasks of extracting the requisite data and then inputting them in the correct form for the relevant audit. At the clinical centres, participants reported numerous practical obstacles to the tasks of data work. The information required to complete the data fields for the audits was typically held in other, usually incompatible systems, so that participants had first to access and process information into a suitable format before they could even begin data entry. Participants reported an excess of data forms and fields, and complained that the same data had to be entered more than once for different purposes. The data-entry forms were reported to be poorly designed or difficult to use, but “upgrades” caused further frustration by requiring users to learn new systems. The within-organization technological support available for these systems was poor; IT malfunctions were frequent and not easily recoverable. The extraction and assembly of the information could not always be completed in time for the data entry schedule set externally by the audit. These mundane obstacles had a powerful impact on clinicians’ ability and willingness to complete data entry.

*If I do a carotid endarterectomy operation then I’ve got the online [Vascular Registry] to fill out, I’ve got a local form to fill out. I’ve often got research paperwork to fill out, I’ve got to write an operating note, and all that can actually take possibly anything in excess of 30 minutes to do all that – and I actually probably only have about 20 minutes between cases sometimes, so it’s actually quite a logistical problem. [Vascular Registry: vascular surgeon]*
*The [completion rate at our centre] is appalling apparently, which is just daft, you know, because it’s just inputting onto computers. […] We do all the difficult bit, we do the diagnosis and we do, you know, the performance data, and we do the staging, but actually just loading it up to [the Lung Audit]? I know why people don’t want to do it, because it’s dull and it’s boring. [Lung audit: radiologist]*

In consequence, a major strategy for both projects was provision of technical support for data collection, entry, and uploading. This was instrumental in securing the improvement in the rates of contribution that occurred over both projects, though in neither case did it resolve problems of data submission entirely.

Mundane interferences – rather than objections on the basis of grand principle – continued to frustrate the collection of data, but a crucial challenge was to address the status of data collection as a form of what Hughes terms “dirty work” ^41^. Data entry was very rarely built into job specifications or organizational charts in the clinical centres, and rarely was it an activity that was directly funded or resourced by organizations. It was thus susceptible to being seen as a discretionary or voluntary activity that was left undone, or fell either to lone enthusiasts or to (sometimes resentful) conscripts.

*Collectively, yes of course it’s important because it’s a countrywide initiative. […] Collecting data is how you then plan forwards, but, and I think that’s the disconnect people have: we have nobody putting data in at all (laughs) unless they’re doing it from the goodness of their hearts. [Lung Audit: respiratory physician]*

Some of the deeper troubles lay in the status of data work as a professional activity, a trouble that Harold Garfinkel anticipated when he noted that professional personnel may question whether record-keeping constitutes “a respectable thing for them to be doing from their point of view” (p194). Highly qualified clinical staff often felt that data collection and input for clinical audits was a menial, tedious task that distracted from the real work of caring for patients. Again, objections were not directed at the principle of audit or its value, nor were they usually linked to anxieties about how the data might be used: rather, it was the practical business of the doing of it that was the trouble.

*We’ve got our own surgical practitioner who does [the data entry] […] except it’s not really his job to be doing all the data entering, it’s his job to be helping teams look after patients. [Vascular Registry: vascular surgeon]*
*Why [are they] sat here doing this boring menial task, when—particularly some of the highly specialist lung cancer nurses—they should be seeing patients? Yet obviously I understand the greater good of the national data and why it’s important. [Lung Audit: radiologist]*

In the sites we observed, the job of data entry tended to settle on someone who was lower down in the hierarchy and less able to resist the imposition of the task. These individuals included the multi-disciplinary team (MDT) coordinators, who were typically administrative staff, with the task constructed as largely a clerical one.

*So everyone says ‘well I’m too busy, I can’t do that’. But everyone says that and to be honest, with us, a lot of it has fallen down to the coordinator to collect it. [Lung Audit: cancer service manager]*

The approach of allowing the task to fall to the lowest level of the organizational hierarchy was, however, problematic. Preparing complex clinical data for submission to the audits required judgment and no small amount of clinical knowledge.

*There’s often a lack of understanding that, you know, they can pay people peanuts or the lowest grade to do this kind of work and whilst maybe that might be possible for some, there’s clearly got to be people with more specialist knowledge involved in directing them to do the right thing. [Lung Audit: oncologist]*

In some centres, the realization grew that pushing the task of data entry too far down could have negative consequences for physicians and others senior in the hierarchy: their performance (and that of their centre) might be misrepresented by poor-quality data, and accordingly the quality of their care could be easily underestimated by external authorities and peers.

*[The audit says we are] below the target for giving chemotherapy in small-cell patients, just under the target, but then of course, you’ve got to look at the numbers. The headache is they realize that actually they haven’t sent the right data in. If the data had been right in the first place, it meant that we were actually above the national target. […] I think what’s clear is that, you know, rubbish in, rubbish out, really. [Lung Audit: oncologist]*
*The MDT coordinator (…) hadn’t added that bit on because she didn’t understand the importance of the squamous-cell carcinoma bit. Once I’d amended that, I think our NOS [‘not otherwise specified’ tumours] rate for last year was only about 10%, which is quite different from how we’d historically been documented, and so I think quite a lot of that is data entry and inaccuracy, rather than poor histopathology. [Lung Audit: respiratory physician]*

The professional leadership of the audits deployed a number of tactics to help bolster the status of what appeared to be dirty work. This included more cultural framing work, characterizing data entry as a professional duty governed by professional values.

*The difference between good and bad practice is acceptance that data entry is part of your job. [Vascular Registry: core project team member]*

A second tactic was the encouragement of a team-based approach to data entry. The Vascular Registry had originally depended on surgeons entering data, usually by inputting to an online database shortly after a surgical procedure, but a team approach to data entry, including nurses and anaesthetists, emerged ^39^. In seeking to promote collective professional responsibility for data entry, the Lung Audit went further, and encouraged what it termed “live” data entry during MDT meetings. Here, the data were entered directly onto the electronic database (often projected onto a screen visible to all team members) during team-based discussions of patients aimed at reviewing their cases and planning their care ^23^. At sites where live data collection worked well, some local actors capitalized on the opportunity to use the technology to improve the flow of meetings, make them more ordered and dialogical, engage all team members, and enhance the quality of decision-making. Done well, live data capture helped to refigure data entry as a higher-status professional activity – and in the process secure accurate, timely, complete data collection.

*It is much easier if it’s one of the medics doing [the live data entry], because they understand the medical terminology really in a more appropriate way. (…) If [a doctor in training] sits at the back it just washes over them, whereas if one of them’s having to type it in, they’re having to say, “What exactly is the staging, what exactly have we said here is the plan?” [Lung Audit: cancer nurse specialist]*

## Discussion

Our analysis of how the data are produced for two large-scale data exercises during an interesting period in their development reveals much about the forces at work in contemporary healthcare, including the enduring role and place of professionalism, the importance of the politics of professional identity in securing change, and the need for renewed attention to the mundane interferences in the achievement of quality improvement goals. The improvements seen in data submission and quality in the two audits during the period we studied, prior to mandatory data collection in either field, were not achieved by defeated professionals subjecting themselves to invasive exercises because they had no choice. Instead, it was their professionally founded character that helped to secure legitimacy and, ultimately, cooperation in the clinical centres. This was achieved by determined strategic action on the part of entrepreneurial and well-respected leaders within the professions: the move towards increased transparency and external accountability was managed in ways that preserved a sense of professional control and ownership, rather than provoking hostility to external intrusion, though the skilful creation of a cultural account ^34^ of data work. The national-level policy context, and experiences in neighbouring clinical specialities, was important. Much policy-level work was going on both front-stage and backstage to improve data collection, and to signal that mandatory data collection was on the cards, but leaders of the Lung Audit and the Vascular Registry also sought to convince their peers that this was a professional obligation, not merely an external accountability requiring nominal compliance. This mission largely successfully achieved, what stood in the way was not principled objection but the mundane frustrations of getting the tasks associated with data collection and entry done. Again, the cultural framing of the activity as a professional duty was important to helping to resolve its status as an abject form of labour. These findings have important implications for other large-scale data exercises, registries, audits and other efforts to create learning health systems to achieve optimal outcomes for patients ^42^.

One way of understanding the turn towards clinical audit as a tool of performance management is to see it as an erosion of professional mandate, in Everett C. Hughes’ sense of the ability “to define – not merely for themselves, but for others as well – proper conduct with respect to the matters concerned in their work” (p287) ^43^. The increasing penetration of accountability tropes into audit might be hypothesized to provoke resistance and hostility by undermining the collegial principles thought to be critical to the organization and experience of professional work ^44, 45^. A key achievement of the professional leaders in our study was that of persuading local clinicians of the importance of collecting and reporting data on quality. They did this in part by appealing to professional values of patient care, and in part by appealing to perhaps less noble (though not ignoble) self-interests, including, for example, enhanced professional legitimacy and retention of a measure of control. A key strength of the cultural account thus created was that professional leaders were then able to use tough tactics that might have been unpalatable had they come from those outside the professional peer group. These findings suggest that professionalizing strategies may have an important role as a means of encouraging commitment to quality and safety efforts. Though sociologists and others have often been sceptical of professional claims that seem aimed at aggrandizement and territory-claiming, more recent work has pointed to the benefits of maintaining a “third logic” of professionalism ^46, 47^ – one that retains a measure of control over work. Importantly, this is not the individual-level autonomy of old, but professional autonomy at a collective level ^48^.

More broadly, our findings point to the increasing interpenetration of two once quite distinct logics: professionalism and managerialism. The increasingly managerial uses to which the two professionally-founded audits were being put might perhaps be understood as a colonization of professional territory by management, but recent contributions ^49, 50^ caution against such a straightforward analysis. This work points instead to the emergence of managerial-professional hybrids that should be seen as neither managerial colonization (hegemony) nor professional co-optation (resistance) ^49^. We see our study as offering some empirical support for this proposition, particularly in its finding of local creativity in use of data and technology for professionally led change interacted with the national-level movement towards transparency.

For those seeking to create and sustain learning health systems, the practical lesson is that allowing professional communities more influence over their own destinies, rather than insisting on external oversight and accountability, may be a more effective long-term strategy for authentic cooperation than unenthused or aggrieved compliance ^51^. Among other things, this means that naming, shaming and blaming by external parties ^52^ is likely to be counterproductive. Such activities, where they seem necessary, may be best done within a peer-led community.

A second lesson for those seeking to conduct large-scale data exercises for improvement lies in our identification of the mundane as a source of trouble. The effort required to produce audit data in the required format, and the lower professional status of those tasked with doing it, had potential to hamper data collection for audits. The wearisome nature of the tasks of data work were, in many ways, a much more potent barrier than grand protests about demands for external accountability. These tasks were a form of what Hughes terms “dirty work”: the kind of stigmatized work that “wounds one’s dignity” (p49) ^41^. Roles at work may be played out in ways that involve a “chronic fight for status” (p53) ^41^, such that, particularly in medical settings, dirty work is often the subject of attempts to “roll” it downhill to lower-status occupations ^53^. But if rolled too far down, to those unqualified in interpretation, it risked the reputations of those higher up who sought to keep their hands clean. The solutions found in these projects relied on highlighting the reputational risks and conferring new dignity on the tasks, refiguring data collection as a professional duty, and creating structures to support collective responsibility for the tasks. Though these strategies were successful in some sites, more institutional support for data tasks is likely to be necessary for long-term success. It is perhaps of note that the Lung Audit’s most recent annual report ^27^ proposes that “all lung cancer MDTs should appoint a ‘clinical data lead’ with protected time to allow promotion of data quality, governance and QI [Quality Improvement]”: the need to promote the value of data, and ensure recognition and status for those administering it, remains a crucial task for those seeking to maximize the quality and thus the utility of audits.

Our study does have some limitations. It is a study of two UK audits only, and the findings may not be generalizable to other settings. The findings apply to a particular period in the evolution of the audits: one that offered a particularly interesting window on non-mandatory data collection, but perhaps one with some unusual features, including the prospect of mandatory data collection in the near future. Nevertheless, increased use of audits, disease registries and other large-scale data exercises for monitoring and improving quality of care is an international phenomenon, and it is plausible that similar challenges of professional alignment, mundane technical challenge, and division of labour will beset programmes in other national contexts.

Our findings thus have important implications for large-scale data collection as the effort to create learning health systems accelerates. Professionally-led activities may offer some significant advantages, and the consequences of mundane obstacles to participation in data collection should be recognized and managed as early as possible.

## Data availability

### Underlying data

The ethical approvals for this study were obtained before policies on data availability were well-established, and even now data-sharing procedures for qualitative research have not been fully agreed upon. The information leaflets provided to participants at the time of the study did not explain the possibility of data-sharing, so their consent for use of the qualitative data for this purpose was not sought. In addition, the data (observations and interviews) are in a form that might allow identification of sites and/or individuals, and so confidentiality might be breached by data-sharing. For confidentiality reasons, no contact details of participants were retained, meaning that a re-consent process is not possible. Given these ethical challenges, data are not being made openly available. Approaches can be made to the principal investigator (Mary Dixon-Woods) or to co-investigator Graham Martin on an individual basis regarding the data. Requests for data access will then be discussed with the ethics committee.

## Reporting guidelines

This paper covers many, but not all, of the requirements of the COREQ guideline for qualitative research. We do not believe all of the COREQ items are relevant or appropriate.

## Consent

We obtained written informed consent to interviews.
