# Supplementary material for: An ethnographic study of improving data collection and completeness in large-scale data exercises
Source: Wellcome Open Res. 2019 Dec 16;4:203. [Version 1] doi: 10.12688/wellcomeopenres.14993.1 (PMC7001749; doi:10.12688/wellcomeopenres.14993.1)
Supplement: Peer review history

# Peer review: Reviewer response for version 1

**DOI:** 10.21956/wellcomeopenres.16355.r37621

**Published:** 2020-02-03

**Authors:** Lorelei Jones

**Affiliations:** 1 School of Health Sciences, University of Bangor, Bangor, UK

Thank you for the invitation to review this ethnographic study of national clinical audits. The study is of two audits, one for lung cancer and one for vascular surgery. The paper describes the challenges of data entry. These include the fact that the work is tedious and time-consuming (yet requires a high level of clinical knowledge), and competes with other priorities, such as patient care. It also documents the strategies of professional leaders aimed at improving compliance with the audit. A key tactic was to align ‘data work’ with professional interests and values. Professional leadership made what might be otherwise seen as undesirable performance management more acceptable and effective in securing cooperation. 

 There is enough detail on how the authors collected and analysed the data to assure me that they understand the approach and methods, that data collection and analysis was systematic and comprehensive, and that their account is credible.

 The paper presents a careful analysis that uses relevant social science theory to explain the findings. The discussion clearly sets out the lessons for improvement programmes, and the implications for broader policy directed at the governance of healthcare quality. The lessons I will be taking with me are ‘the importance of the politics of professional identity in securing change’, ‘the need for renewed attention to the mundane interferences in the achievement of quality improvement goals’, and that ‘allowing professional communities more influence over their own destinies, rather than insisting on external oversight and accountability, may be a more effective long-term strategy for authentic cooperation than unenthused or aggrieved compliance’. I will also be coming back to this paper in my work because of the theoretical contribution it makes in relation to the role of professionals in healthcare governance.

 I suggest the authors provide a reference for improvement work on the ‘front stage’ and ‘back stage’. These are helpful concepts for understanding the politics of improvement work and a reference would allow the reader to find out more. 

 The findings are clearly communicated by the very high standard of academic writing. An enjoyable read.

I confirm that I have read this submission and believe that I have an appropriate level of expertise to confirm that it is of an acceptable scientific standard.

# Peer review: Reviewer response for version 1

**DOI:** 10.21956/wellcomeopenres.16355.r37620

**Published:** 2020-01-30

**Authors:** Lesley Gotlib Conn

**Affiliations:** 1 Sunnybrook Research Institute, Toronto, Canada

Thank you for the invitation to review this article, “An ethnographic study of improving data collection and completeness in large-scale data exercises.” This ethnography examines the social complexity involved in achieving data completeness and data quality in large-scale clinical registries. Drawing mainly on professionalization theory, the authors explore the social processes involved in achieving improvements in two UK registries – the National Lung Cancer Audit and the National Vascular Registry – vis-à-vis a quality improvement programme. Through interviews, non-participant observations and document analysis within the two projects, the authors articulate two significant influences on clinicians’ voluntary participation in clinical audit work: professional leaders’ strategies to promote the collective professional responsibility and value of data collection as an important form of self-governance; and, a reconfiguration of the perceived mundane low-level data collection work as a critically important professional duty. The authors suggest that in order to get clinicians’ to truly cooperate in such data work, strategies are needed that preserve their sense of professional control and ownership over the audit process and a sense of specialized knowledge and responsibility in the production of data itself. 

 The article is well written and effectively tells the story of large scale clinical audits and their known challenges. Through the lens of professionalization theory, the analysis offers interesting and novel insights that are surely transferable to other clinical environments where registry work is part of daily clinical routine. The findings certainly resonate with my own research in a trauma quality improvement programme where concerns about data collection and quality, and the need for effective leadership strategies feature prominently in clinicians’ narratives about program success. The study is well situated in the current literature. The choice of ethnography is ideal and appears to be well executed. Could the authors offer further detail in the methods with respect to the following: how many centres participate in each audit; of these, how many centres/participating sites were represented in the ethnography; and, where in the participating centres did the observations take place? These details would help the reader appreciate the scope of the study and the specific settings from which the conceptual insights were developed, e.g. were these MDT meetings, administrative meetings, shadowing clinicians?

I confirm that I have read this submission and believe that I have an appropriate level of expertise to confirm that it is of an acceptable scientific standard.
